# Strengthening woman-centred care for pregnant women with female genital mutilation in Australia: a qualitative muti-method study

**DOI:** 10.3389/fgwh.2024.1248562

**Published:** 2024-01-16

**Authors:** Sabera Turkmani, Angela Dawson

**Affiliations:** ^1^ Faculty of Health, University of Technology Sydney, Sydney, NSW, Australia; ^2^ Maternal, Child and Adolescent Health, Burnet Institute, Global Women’s and Newborn Health, Melbourne, VIC, Australia

**Keywords:** woman-centred care, patient-centred care, female genital mutilation (FGM), guideline, women, marginalised and vulnerable groups, maternity care, midwife

## Abstract

Woman-centred care is a collaborative approach to care management, where the woman and her health provider recognise one another's expertise and interact based on mutual respect to provide adequate information and individualised care. However, woman-centred care has not been fully achieved, particularly for women who have experienced female genital mutilation in high-income countries. A lack of clear guidelines defining how to implement woman-centred care may negatively impact care provision. This study sought to explore the quality of point-of-care experiences and needs of pregnant women with female genital mutilation in Australia to identify elements of woman-centred care important to women and how woman-centred care can be strengthened during consultations with health professionals. This multi-method qualitative study comprised two phases. In phase one, we conducted interviews with women with female genital mutilation to explore their positive experiences during their last pregnancy, and in phase two, a workshop was held where the findings were presented and discussed to develop recommendations for guidelines to support woman-centred care. The findings of the first phase were presented under three distinct categories of principles, enablers, and activities following a framework from the literature. In phase two, narrative storytelling allowed women to share their stories of care, their preferences, and how they believe health providers could better support them. Their stories were recorded visually. This study highlights the importance of a comprehensive approach to woman-centred care involving experts, clinicians, community members, and women in designing education, tools, and guidelines.

## 1 Introduction

Woman-centred care is an integral part of high-quality maternity care. Woman-centred care is a collaborative approach to care management, where the woman and her health provider recognise one another's expertise and interact based on mutual respect. The focus is on providing adequate information and continuity of care that considers a woman's individual needs, beliefs, values, and right to make her own choices (1, 2). Putting people at the centre of care improves healthcare collaboration, satisfaction, communication and safety and empowers consumers (3, 4). Woman-centred care is acknowledged by the International Confederation of Midwives (ICM), the World Health Organization (WHO) (5) and other United Nations (UN) agencies in reproductive health and maternity care guidelines and recommendations (6). In Australia, consumer-centred care emphasises the importance of leadership and person-centred approaches throughout the clinical care journey and focuses on involving consumers in planning, implementing, and evaluating their care (7).

Female genital mutilation (FGM) is a cultural practice involving the total or partial removal of external female genitalia for non-medical purposes. The practice has no health benefits and can negatively affect a woman's physical and psychological health, resulting in obstructed labour and perineal trauma (8). Migration from countries where FGM is traditionally practised has meant that health professionals in high-income countries (HICs) increasingly care for affected women and girls.

Despite progress, woman-centred care has not been fully achieved for migrant populations in many HICs due to language barriers, low health literacy, and a lack of health provider knowledge and cultural awareness (8, 9). Research has found that women affected by FGM do not always receive satisfactory maternity care that is tailored to their needs, values, and cultural preferences (10–12). Women affected by FGM report the importance of appropriate communication with providers for a positive care experience. Women have described communication difficulties resulting in isolation, a lack of involvement in care decision-making, mistrust, and reluctance to disclose their FGM and related issues (12, 13). Studies also show that health providers lack cultural competence and the technical knowledge required to deliver quality care to these women (10, 12, 14, 15).

A scoping review by Dawson et al. (16) reviewed 124 FGM-related tools and guidelines from six high-income countries and found that only 10% of those guidelines addressed the principles of woman-centred care. A lack of clear guidelines defining how woman-centred care can be achieved may negatively affect health care provision. The review found that clinical guidelines on FGM that indicated the need to involve a woman in her care were limited mainly to obtaining consent (8, 17, 18). Guidelines lacked adequate acknowledgement of the importance of promoting other aspects of shared decision-making, such as supporting women in special circumstances (e.g., refugees and those experiencing domestic violence) and women's individual needs and preferences (19). Other guidelines focused on the behaviour and interactions of providers rather than developing a collaborative relationship between women and health providers (20–22).

Women are the primary users of maternity care services, and their unique experiences and perspectives can provide valuable insights into the design and implementation of care guidelines (23, 24). Involving women in the development of guidelines can ensure that they are tailored to meet their individual needs and preferences, leading to better outcomes and higher care satisfaction rates (24). While there are general efforts to increase women's participation in guideline development through initiatives such as patient and public involvement (PPI) (25), there is little evidence of the direct involvement of women who have experienced FGM in developing or co-designing guidelines or tools (16). This study sought to address this gap by exploring the quality point of care experiences (refers to the direct interactions between women and healthcare providers at various stages of their care) and needs of pregnant women with FGM in the Australian health system to identify what elements of woman-centred care were important to women and how woman-centred care can be strengthened during consultations with health professionals. We aimed to provide recommendations to develop woman-centred care communication guidelines (statements, based on available information and best practice that support health professionals to manage specific issues, situations or circumstances) and tools (set of instructions to help health professionals enact such guidelines) to assist health professionals in delivering quality care to women with FGM.

## 2 Methods

This multi-method qualitative study comprised two phases. The first phase involved interviews with women with FGM to understand their positive point-of-care experiences with clinicians during their last pregnancy. The second phase consisted of a workshop where the findings of the preliminary analysis were presented and discussed. This allowed women to confirm, add to, or modify the findings and identify key recommendations for developing a tool to support woman-centred care.

### 2.1 Phase one

In phase one, a qualitative explorative study was undertaken, and data was collected via semi-structured individual interviews with women. A content analysis of interview data was performed to identify women's maternity woman-centred care interaction experiences and needs. The study participants were all from Sydney, Australia. We recruited purposively with the support of a trained bilingual community worker who was part of the research team and was known to and trusted by migrant community members. The participant inclusion criteria were women with FGM who had migrated to Australia and had a birth in the last two years in Sydney or were pregnant. Due to COVID-19 pandemic social distancing restrictions, we could not conduct face-to-face interviews with women. All interviews were undertaken online using the Zoom platform. The interviews were conducted in English, lasted approximately 45 min, and were audio recorded and transcribed verbatim. All study participants received an information sheet, and their questions were answered before the interview. We reimbursed women for their time with a supermarket gift certificate. Written and verbal consent was obtained from all participants at the beginning of the interviews. Ethical approval was obtained from NSW Health AU RED HREC/19/WMEAD/29.

The data were analysed deductively, as per Kyngas et al. (26). We employed the model of patient-centred care identified by Scholl et al. (27) to code the data according to the three domains of this patient-centred care: principles, enablers, and activities and 15 dimensions across these domains (see Table 1 for more details).

**Table 1:** The domains and dimensions of patient-centred care Scholl et al. (27).

Domains/Dimension	Description
Principles 1. Essential characteristics of the clinician 2. Clinician-patient relationship 3. Patient as a unique person 4. Biopsychosocial perspective	1. A set of attitudes towards the patient (e.g., empathy, respect, honesty) and oneself (self-reflectiveness) as 2. well as medical competency partnership with the patient that is characterised by trust and caring 3. Recognition of each patient's uniqueness (individual needs, preferences, values, feelings, beliefs, concerns and ideas, and expectations) 4. Recognition of the patient as a whole person in his or her biological, psychological, and social context
Enablers 1. Clinician-patient communication 2. Integration of medical and non-medical care 3. Teamwork and teambuilding 4. Access to care 5. Coordination and continuity of care	1. A set of verbal and nonverbal communication skills 2. Recognition and integration of non-medical aspects of care (e.g., patient support services) into health care Services 3. Recognition of the importance of effective teams characterised by a set of qualities (e.g., respect, trust, shared responsibilities, values, and visions) and facilitation of the development of such teams 4. Facilitation of timely access to healthcare that is tailored to the patient (e.g., decentralised services) 5. Facilitation of healthcare that is well coordinated (e.g., regarding follow-up arrangements) and allows continuity (e.g., a well-working transition of care from inpatient to outpatient)
Activities 1. Patient information 2. Patient involvement in care 3. Involvement of family and friends 4. Patient empowerment 5. Physical support 6. Emotional support	1. Provision of tailored information while taking into account the patient's information needs and preferences 2. Active involvement of and collaboration with the patient regarding decisions related to the patient's health while taking into account the patient's preference for involvement 3. Active involvement of and support for the patient's relatives and friends to the degree that the patient Prefers 4. Recognition and active support of the patient’s ability and responsibility to self-manage his or her disease 5. A set of behaviour that ensures physical support for the patient (e.g., pain management, assistance with daily living needs) 6. Recognition of the patient's emotional state and a set of behaviour that ensures emotional support for the patient

First, all the transcripts were uploaded into NVivo software. After initial coding, the authors checked the transcripts against the codes, and all discrepancies were discussed and resolved. Consequently, themes are captured based on the dimensions of each domain of women-centredness.

### 2.2 Phase two

The second phase involved a two-hour workshop designed to build upon the findings from phase one and incorporate insights gained from women's narrative storytelling. The workshop's primary objective was to collaborate with women to elicit their views and inputs on the results of the first phase and how they could be translated into practice to improve the quality of care at the point of care. We employed a graphic artist to visually capture women's preferences and values expressed in these stories during the workshop that can be used to develop a tool to enhance woman-centred care for FGM-affected women in Australia. In addition, notes were taken of the stories women told, and any notes or drawings they produced were collated.

A bilingual community worker assisted us in purposively recruiting participants through community centres. We distributed flyers and displayed these to inform women about the study and study participation conditions in the community centres. The eligibility criteria included women aged 15–45 who have undergone FGM and received maternity services in Australia within the last 3–5 years and could communicate in English. The workshop was held in a training room at a community centre, which was conveniently located near most women and accessible by public transport. Women were given a supermarket gift card to reimburse them for their time and travel. Before the workshop began, the participants were asked to read the study information sheet and provide written consent to be involved in the study.

The workshop included various interactive activities such as presentations, drawing, roleplay, and group discussions that were carefully selected to engage women and empower them to take an active role in the session. The facilitators (ST & AD) presented the findings from the first phase of the study and highlighted key themes and issues that emerged. The women were then invited to share their thoughts, insights, and personal experiences related to the themes presented. Roleplaying activities allowed the women in pairs to explore different scenarios and provide solutions to improve woman-centred care. Women were encouraged to draw or note ideas using pens and poster paper. Women gave feedback on what had happened in their role plays, and the group discussed issues raised and ways forward to improve interactions with health providers. After the workshop, the women were invited to review the initial version of the visual recording and provide feedback to ensure that it had captured their key views and preferences on the elements of woman-centred care that were most important to them. Feedback was incorporated into the graphic, along with notes or materials produced during the workshop.

## 3 Findings

### 3.1 Phase one

We interviewed 13 women affected by FGM in New South Wales, Australia. The study participants came from five countries where FGM is commonly practised: Somalia (two women), Sudan (five women), Sierra Leone (three women), Egypt (two women), and Ethiopia (one woman). Their ages ranged from 26 to 44 years old, and all of the women had given birth within the past three years. Three of the women were pregnant at the time of the interviews. The majority of the women had undergone either type I or II FGM, except for those from Sierra Leone and Somalia who had experienced type III FGM. All of the women, except one primiparous woman, had given birth multiple times.

**Figure 1:**
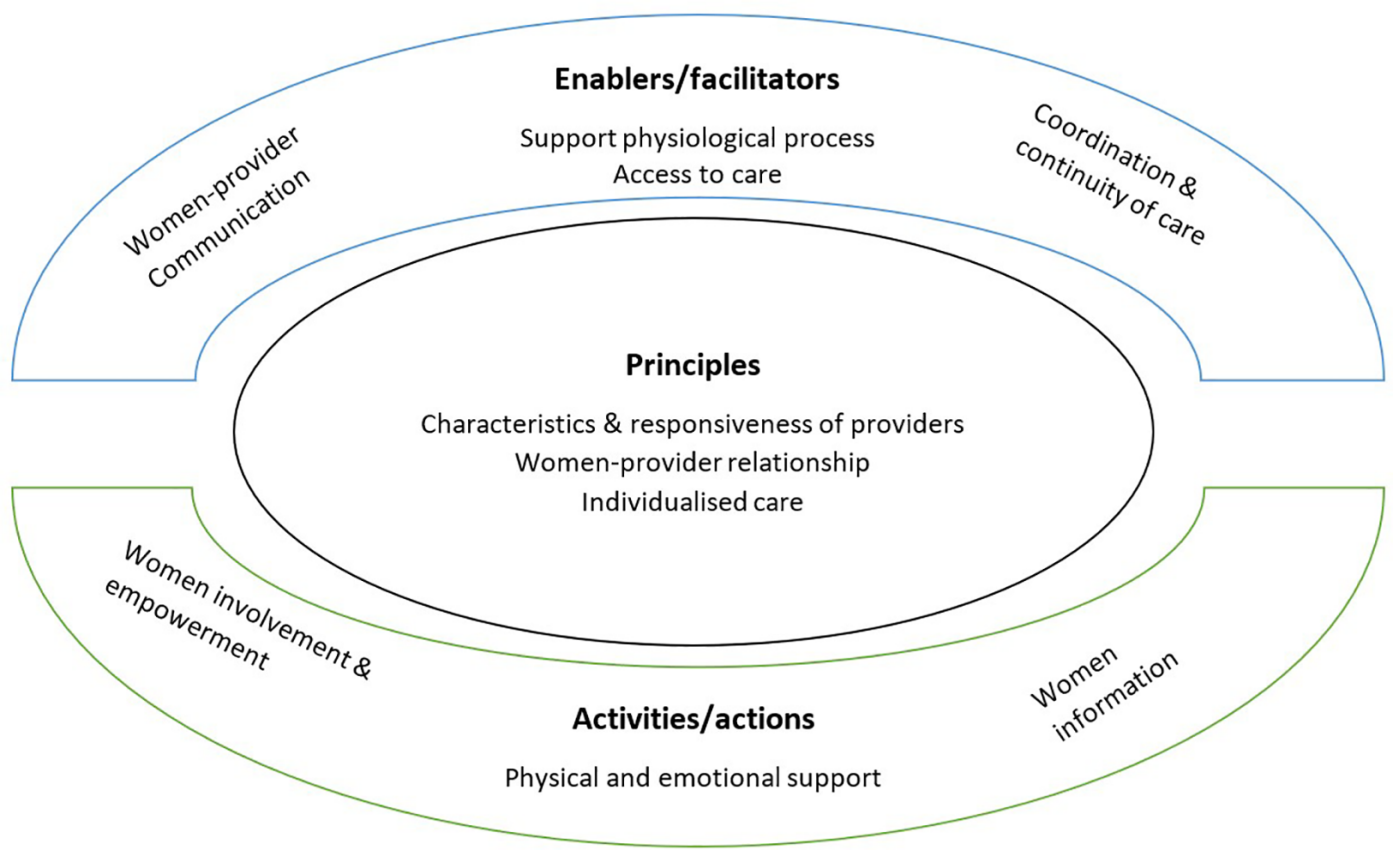
Foundations of women-centred care based on women's views.

Our content analysis of the interview data [based upon the *a priori* domains outlined by Scholl et al. (27)] resulted in the identification of ten themes that are grouped under the three domains’ principles, enablers and activities’. Themes under these domains based on the dimensions were modified to reflect the data. These included characteristics and responsiveness of health providers, women-providers relationship, individualised care, women-provider communication, support of physiological process, access to care, coordination of care, physical and emotional support, women involvement and empowerment, and women’s information. In the following sections, each theme is elaborated upon in detail (see Figure 1).

#### 3.1.1 Principles

Under this domain, four themes were identified, including ‘characteristics of the health providers towards women, individualised care, physical and emotional support, woman-providers relationship, and support of physiological process’. Elements concerned with the whole person in his or her biological, psychological, and social context permeate these themes.

##### 3.1.1.1 Characteristics and responsiveness of the health providers

This theme pertains to the qualities that women expect their healthcare providers to possess. Participants highlighted the importance of kindness, compassion, respectfulness, responsibility, commitment, and cultural competence. The majority of the women in our study had interactions with midwives during their pregnancy and childbirth. For some women, their initial point of contact was their General Practitioner (GP), who then referred them to a midwife.

The most cited characteristics concerned how the women were greeted and how the midwife responded to the women’s concerns, highlighting the importance of respectful and culturally-sensitive care in midwifery. Women emphasised midwives understanding of cultural practices and backgrounds and their knowledge about different health concerns as it can make them feel comfortable and supported. The following quote showcases these findings:

*..always midwives smiled, they greet me well and they welcome me … greets me as well. She examines my baby and examines me, takes the high blood pressure test and then she wrote—if I need to go to the ultrasound or she wants to get the blood test or she wants to do any test, the midwife just writes it for me. During the meeting with her midwife, she asks a lot of questions about pregnancy and she asks her about her health and her baby then everything. (W2)*

Women identified midwives whose experience and training had enabled positive attitudes and responsive care.

*….all through the three pregnancies, when I went to antenatal clinics at both X hospitals, where I was living at that time and X Hospital, the midwives did ask those questions of whether you've done it because I think obviously they have the understanding that people—it’s very common within people, Middle Eastern and African backgrounds. (W5)*

Another woman emphasised her belief that midwives had undertaken” their own research and educated themselves to cater to those people's needs. So—but yeah, that helps a lot."(W1)

While some women stated that their midwives were not so aware of the risks and implications of FGM, and referred them to their GP, one woman noted that some providers might not be as open-minded and understanding about cultural practices, such as FGM. This lack of knowledge may result in judgement or criticism.

*Because I think she’s [midwife] very understanding of different cultures and different people from different backgrounds and stuff and she’s very respectful, whereas I think some people are more closed minded about a few things. So, like if you tell them oh, I've been circumcised, some people are like oh that’s so horrible, what a horrible culture, oh what a horrible country to live in and blah, you know? People don't choose you know, certain situations, so you've got to be more understanding when it comes to personal things that happen to people. (W3)*

##### 3.1.1.2 Woman-providers relationship

The findings indicate that relationships with midwives are an integral part of care. Women described midwives who were understanding, friendly, and attentive to their needs as new mothers. Women reported that midwives who built strong, supportive and collaborative relationships with them helped reduce stress and anxiety and ensured a positive outlook during pregnancy and childbirth. One woman described this relationship and the integration of clinical and non-medical care.

*I have beautiful relationship with my midwife, I feel like I'm so comfortable and I'm so open with her, like I can tell her anything, because she already knows everything that I went through. She was there with—when I first got my reverse I went to her and she—yeah, I feel really comfortable with her and I really love her support. She’s an amazing midwife, so yeah. (W3)*

The women emphasised the importance of clear communication with their midwives and receiving reassurance and advice. One woman noted, “*I felt safe talking about it to midwife*” indicating how her midwife has created a comfortable and safe space to share information about her FGM*.* Showing respect and actively listening by paying attention to women's concerns and questions were indicators of a good relationship, according to the participants. For example:

*Yeah, she’s [midwife] very friendly and she always talk with me clear words that can help me for understand. Yeah, she’s really unique midwife and yeah, she’s really good. She always advise me, yeah. She always told me to not worry about anything. You will be okay, yeah. She always listened for any question I ask of her. She always respects my view. Talk to me with laughing or smiling and say hi, Maha, how are you? (W4)*

Several women discussed the importance of a relationship with a female provider and having midwives with similar cultural backgrounds that may enhance trust between healthcare providers and patients.

##### 3.1.1.3 Individualised care

Women appreciated their midwife's expertise in managing specific types of FGM and her willingness to provide individualised care. One woman's outlined how her midwife had adapted her practice even in challenging circumstances.

*(My) midwife said that it is hard for, my type of FGM to get a normal birth. It’s hard, but because my [midwife] is specialised. My midwife was specialist for that type of FGM and could help me. She [midwife] managed a lot of my type of FGM [type3] and she told me that she’s going to open it. She’s going to open a little bit of the area for the head of the baby to come out and then. Yeah, my midwife was very good after the day of her birth, midwife called me for follow-up. (W12)*

Another participant spoke about the efforts of the medical team to ensure that she received supportive care tailored to her needs.

*All very comforting, they offered me counselling and when I was pregnant with my daughter and they saw and when they did the assessment and stuff, the first time, they offered me counselling and you know they gave me so much support, it was absolutely overwhelming. It was beautiful support and then I remember the first time after I had my daughter and then the doctor said to me, oh we can reverse it for you. I cried in the assessment room, because I was like you can reverse it so I can be normal again? I was just so really, really emotional– and then the doctors were so comforting and oh my God, they were such beautiful people and then I had that procedure, like reversed the whole thing and then yeah, it was—oh my God, it was beautiful. [Laughs] (W3)*

#### 3.1.2 Enablers

Under this domain, the themes are focused on women-provider communication, support of physiological process, access to care and coordination and continuity of care. Together, these enablers can help create an environment in which women feel comfortable discussing their health concerns with their healthcare providers and feel that their needs are being heard and addressed. Below is an elaboration of each of the above mentioned enablers.

##### 3.1.2.1 Woman-provider communication

Women found the communication with midwives more relevant than GPs because the discussion with midwives was more focused around pregnancy-related issues, risks, and procedures.

*Going to GP it’s, like you know like it’s a general, like general questions. Yeah. They[GPs] just then send you to the ultrasound and the x-ray and the stuff like this, but it’s not like, the midwife because the midwife they talk with you more and more about the pregnancy related issues and what you're going to do and what are the risks—about the operation and the stuff like this.(W6)*

One woman found that her midwife’s fun way of talking about confronting topics, such as FGM, helped to alleviate stress and increase her confidence in communicating her issues.

*Just like, my midwife—like, she was good to make fun anyway. Like, saying something that’s funny … you know, something that only just take your mind away from thinking about something bad you know. I like her for that. It’s good because we're already stressed with our pregnancy. we don't sleep very good. that’s all we want you to do. Just to talk to us nicely and give us confidence that why you are going to do this or that. (W2)*

Some women found that prompting questions before difficult conversations were beneficial in preparing them for the question and making them feel more comfortable.

*think it’s the fact that I remember at [name of health service] they prompted me well before of to let me know that difficult questions are coming and some of those will make me feel uncomfortable. So I think that that is important, especially when you're having difficult conversations, letting the person know beforehand, before even you start those conversation is important. (W5)*

However, despite a largely positive experience, one woman indicated the need for her providers to explain more clearly what was involved in procedures.

*They actually, most of the midwife they are talking nicely, they are doing the same, they're talking nice to me, but my main issue, is not about how they are talking and behave, because they all behave nicely and they're lovely…But my main issue with the midwife and the hospitals, is that do not tell us about what they are going to do, what they going to have? They (midwives) need to be clear and talk to you about anything they're going to do and they're asking me more and what do you need and what do you want, because I feel most of the midwives they have like the, they have a task or duty they have to doing with you and that’s it.. (W6)*

Some women felt that midwives did not give them clear answers or enough information to make informed decisions about their options for normal childbirth.

*they (midwives) just every time I would go into the hospital for my antenatal appointment, they would always just have a look and then they would be just like nodding their head. They're like I don't even know how this is going to be possible, so then what they were thinking about, one of my options were that they open it up before I have my child, so that I can try to have a natural birth, because they were like otherwise, if you go into labour the child’s going to be blocked because it’s fully closed. (W3)*

##### 3.1.2.2 Supporting physiological processes

Women valued the support of midwives in facilitating normal childbirth but also recognised the importance of safety and interventions when necessary. It highlights the need for midwives to communicate clearly, provide sufficient information to women, and consider individual circumstances and preferences in providing care during childbirth.

*the baby was not in the right position and they told me we're still going to give you a chance, and they [midwives] were waiting for me to get a normal birth, until I felt that I was not comfortable I she couldn't handle it anymore. So they sent me to the operation- I asked them to go for the caesarean. So, they sent me for the caesarean section. (W1)*

Several women talked about deinfibulation, and one woman noted the importance of supporting this during the antenatal period rather than during birth.

*Then they said to me, what we'll have to do is we'll probably have to cut it and then I was just scared of getting circumcised again, so I was like no. I was just like no, that’s just too much trauma, I'll just do the c-section. Because it’s easier if you don't want to go through the pain and stuff again. Because imagine being pregnant, heavily pregnant, having to think about contracting and giving birth to a child and then also having a procedure before you give birth and having to heal your body. It’s just it was too much trauma…(W3)*

##### 3.1.2.3 Access to care

The findings under this theme shed light on the availability of the various care options for women affected by FGM, such as reconstructive surgery, mental health support, use of technology and counselling services. These interventions were found to be valuable in improving women's physical and emotional wellbeing and in addressing some of the complications associated with FGM. One participant shared a positive experience of using telehealth technology to check on her baby after delivery during the pandemic. The participant described this experience as “really good” and appreciated the midwife's ability to advise her even when they were not physically together.

*During the Pandemic everything was on phone. After delivery when I came back home [from hospital] and then the baby was shaking in her hands, and I didn't know what’s happening. So, I called midwife and asked her, the baby’s shaking. Then she said okay, because I can't see her now, whenever she starts doing this, just film her, do a video and then we can check it. It was really good to because she [midwife] could see the baby and advise me what to do.(W10)*

Women appreciate the access to different services that address their general and individualised needs during pregnancy and childbirth. Access to culturally sensitive care, thorough regular checks, and early diagnosis and monitoring of health issues were assuring factors to women for positive maternal and infant health outcomes.

*The good thing is they [midwives] came straightaway because I always heard from other women, my friends that they didn't come in the first week. Some people, they visit them in the second week. Me, I was lucky, they came two days after I came from the hospital. Yeah, they just check the wounds and what is it, the wound, wounds? For me, yeah, they check if it’s all good and check the bandage. They sent me a letter to come after three months to do the test for the diabetes and what to do if I don't want to get pregnant again, what to do and all these things.(W10)*

Women considered access to reconstructive surgery as an essential option to improve their quality of life. For instance, one woman mentioned that access to such services was important to try to resolve the adverse effects of FGM.

*Then obviously, when it comes to giving birth naturally, that wasn't an option for me because of the way that things healed [FGM] and how—I've had to have several surgeries to try to get it reversed in Australia. Yeah, I done it, yeah, before I had my daughter, I had a procedure to try to get it reversed. Then after I had my daughter, then I had to have another procedure, so I've had a few procedures to try to undo it completely. But I don't know, it will never be the same obviously. (W3)*

##### 3.1.2.4 Coordination and continuity of care

Having a consistent and supportive midwife can make a significant difference in how women feel about their care. In addition, being with a known midwife or having midwives who understand and respect cultural differences can help women feel more comfortable and empowered them.

*During my antenatal clinic, [the midwife], I had her for both my pregnancies, and she was my midwife. I think from the midwife and from the maternity care—and I think they [midwives ] do everything—I think the GP like, are there for the referral part as the only thing—maybe if the midwife knows a particular doctor that’s more experienced with FGM, they could recommend that to women. But—because not all GPs know about it[FGM] and not all of them are familiar with it actually. So, I think just having the right team around the woman, the woman, would be able to get the best care. (W13)*

Regular post-natal visits and information were noted as important facets of care.

*midwife come like two times at home and she say if anything happen you can come to the hospital—if anything happen to me or the baby, and she told me, and she gave me a list of the, like the childhood centre, they're caring for the baby for the weight and immunisation, first immunisation, stuff like. Yeah, she say she can continue with me on the phone, but if I need any help I can go to the nearest child health centre.(W6)*

#### 3.1.3 Activities

The findings under this domain highlight the importance of engaging in activities that promote physical and emotional support for women, involving women in decision-making processes, and sharing information with them. Some specific activities that can be taken include:

##### 3.1.3.1 Information

Women talked about sharing knowledge and critical information and how it should be tailored based on women's needs, and delivered in a supportive and non-judgmental environment. Some women confess that they did not fully understand the side effects of FGM till a midwife provides them with comprehensive information about potential risks. One woman said:

*But I know I went through it [FGM] and I had pictures of—because it was something that was celebrated back home, so I saw pictures of it. But as a child I didn't have much experience to know whether, I had been undergone or not. [Laughs] So it was interesting that the midwife was talking about all these effects when you get that done, all the side effects, the pros and cons, especially when you're pregnant. She provided that information and that was a surprise for me, because I didn't even know when you got this done, these were the side effects. (W5)*

One woman appreciated the way her midwife provided relevant information on FGM without overwhelming her:

*what I like in pregnancy is information. I'm very—I'm an organisation freak. So I like as much information as I can get from the midwife in terms of—like, especially with my first child, when I had no idea what to expect, she gave me—the things I [felt] overwhelming on the internet and stuff, they tell you that you need this and you need that. So, she gave me a little breakdown of what I needed to pack for my hospital bag, what to expect. Just information. I like information. (W13)*

While some women believed that more information about FGM needs to be developed, others stated that there is plenty of information about FGM for women affected by FGM, but information for the wider community is needed.

*I think there’s not a lot of education around it [FGM]. Like, if you're in the community that’s affected by it, obviously you're surrounded by it. But in the greater community that no one really knows what it (FGM) is—it’s like it’s almost as if it’s a disease. Like, when people find out, it’s like, oh, I'm so sorry. But I just think information for the wider community to know what it is and how it can affect people and like because it can affect people’s self-esteem. So, I don't think we needed that—we need the added pressure of people almost looking weirdly at it. You know what I mean? (W13)*

##### 3.1.3.2 Women’s involvement and empowerment

The findings under this theme suggest that when women are actively involved in the decision-making process about their care, they feel empowered and can take responsibility for their care. However, the study participants had different experiences about the extent to which they were involved in decision-making and stressed the importance of receiving guidance from their healthcare providers to make informed choices.

*They have given me choices and for each sometimes they asked me if I am happy to go for that. They also told me what is going to happen, what are the good things and bad things on this one…then I have to choose whatever suits me. But they don't do that always, sometimes they get a decision and they tell me like just you must do this. Even I didn't know if it was good for me or not. (W1)*

Women commented on feeling empowered to express their needs relating to their family’s involvement and communicated instructions to her midwife.

*With the ones that I went to, yes, because they were like a refugee clinic at The [name of Hospital]. So they already knew, because even when I went into labour, I had a Lebanese midwife that was working with my midwife—I said to her, my in-laws are going to come, but there’s a lot of them and I don't want them to see everything, but it’s rude in my culture for me to tell them to get out. So, I said, can you please just tell them there’s only two people? because I don't want them to see anything and they listen to me? (W13)*

Some women felt overwhelmed or uncertain about making decisions regarding their healthcare and may defer to the expertise of healthcare providers. One woman said she trusted health providers’ judgment over their own when it comes to making decisions about their health and medical procedures.

*if it can happen, the decision like I said to you, sometimes you can't decide what is good for you because you're not the doctor. They're the doctor. They should decide. You have to have this because if you have this, this can happen. If you don't have this, this is going to happen. They leave it to women and then they say, yeah, you decide what you want. Well, I came to you so you decide for me because you are the doctor, not me.(W10)*

Women emphasised the role of health providers to provide timely information and help them make informed decisions; however, sometimes this was not possible.

*I wasn't involved in decision-making because I tried to have my baby normal delivery but in the end, there is no choice, they have to take me to the Caesarean section so she just, yeah they just told me that have to do it and I did it, I don't have any choices because the baby was overdue already. (W8)*

Women mentioned that the midwives referred women to a doctor who could provide more information and make the final decision.

*They [midwives] said they need to open (FGM), they need to cut more to get my baby out and I got bleeding and stuff like this, you know. So I was worried about the second time [being cut] and I was asking for cesarean. I told them, because like I feel complicated from the normal delivery. They talked to me, they bring my file and they try to explain what happened and then they said I need to see the doctor, the doctor who will decide what I need….They [midwives] answer what they know, but most of things like that, if I am asking about the big issue, like when I'm asking what about if I need to get like a caesarean, not normal birth, they [midwives] say no you have to—they don't take the responsibility. They say when you meet the doctor, the doctor will tell you more and will explain to you. (W6)*

One woman also acknowledged how her midwife respected her choice and empowered her to make her own decision.

*You know how they [midwives] give me the plan, whatever, how I want your labour day to go? So, I didn't want an epidural or anything like that. So, the midwife,… she’s like, you said you want that [option], but now wait till you're in pain and then I'll ask you again. So, on the day when I was going through labour, she had asked me and I said the same thing, although I was in a lot of pain. Then my partner said no to her, but she [midwife] told him—she’s like, I'm not talking to you. This is about her. (W13)*

##### 3.1.3.3 Physical and emotional support

Women shared positive stories of health provider support that highlighted compassionate care and the positive impact on their physical and mental health and recovery.

*the way people talk to you they make you pleased, even if you are upset or you stressed but the way some people talk to you they make you feel good. You know what I mean? So they talk to me like, ‘don't worry you'll be fine’, ‘I know this is not easy’. So the [way they] see me but for me is a midwife. For me I have no idea so they watch me and say you don't look good. You don't look good. (W2)*

Women also emphasised on non-verbal interaction, such as holding or offering a chair or water, as an indication of kindness and emotional support in a potentially stressful time.

*my midwife will come stand closer to me[ask me] ‘You want to hold my hands? You want me to bring chair so like, we'll try and go inside the room when they check the baby, the baby is doing well heart beating. They will say to me sit down there. Have a rest. Do you want me to bring this? You want to drink a water? Which was very, very good, they are talking to people nicely. (W2)*

Women often appreciate emotional support and reassurance words by providers. Words such as ‘relax, calm down, don't worry, no problem’ give them a sense of safety and comfort to express their concerns and fears.

*Because you feel like you're going through it alone, until somebody says oh, we know what you've been through, we know what your options are, we can help you out in that area. That’s the biggest relief that you could get from your doctor or midwife. You feel so supported when someone says that to you. (W3)*

Offering practical assistance, such as guidance on breastfeeding, caring for the baby, and managing postpartum recovery, was also considered as helpful.

*She (the midwife was) helping me with my baby for breastfeeding, she stay with me like two hours, tell me how to change him, she was talking with me, even she know I got another girl, but she was helping like it’s my first time to give birth, because I was caesarean and I have a wound. She takes the baby for me. She holds it and she told me how to hold it and how to get care of my back, because I have a wound and how to clean it, how to clean it.(W6)*

Connecting women to additional resources like counselling or support groups can help them to feel empowered and supported throughout their pregnancy and beyond.

*So, she (the midwife) put me in touch with a counsellor at the hospital. They were talking to me because I was very depressed at that point… She called me every week because my appointments would be at the beginning, once every month and then the further I got, the less time I needed to be away from her. She gave me a welcome pack that had nappies and baby food and little samples and stuff in it. It was honestly—it was better than anything I could have expected. I was deciding whether to go private or not. But I'm so glad that I went with them, because my experience was amazing. (W13)*

### 3.2 Phase two

Eleven women with FGM from Sudan, Somalia, Egypt and Ethiopia participated in the workshop. Women confirmed the findings from the interviews that were presented to them using deidentified quotes. Many women shared stories about their experiences, adding to the richness of the themes.

Of their own accord, women shared their experiences of undergoing FGM, highlighting its negative impact on their lives (28). The discussion session allowed some women to talk about their traumatic experiences and the various coping mechanisms they employed to deal with the aftermath of FGM. Some women disclosed that they had not discussed their pain with others or sought counselling services to cope with the trauma. Others described how they had become aware of their triggers and made conscious efforts to avoid them.

**Figure 2:**
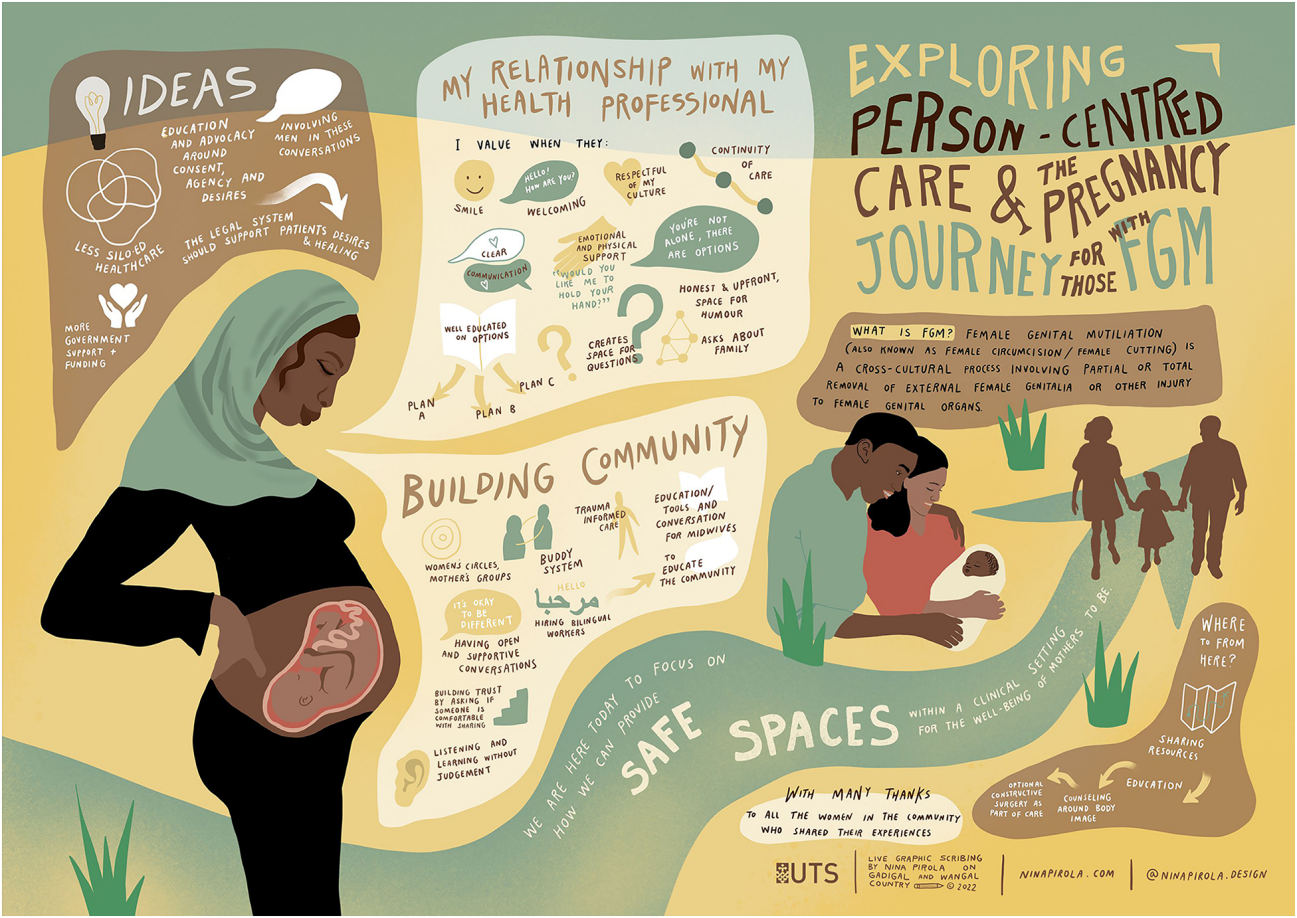
The visualization of women's need and their recommendations for improving the quality of care and establishing an enabling environment for FGM prevention.

The needs of women and their recommendations for improving the quality of care and building an environment for support and prevention were captured visually (see Figure 2). Women outlined qualities and behaviours they had experienced and expected of their health providers, elements necessary in their communities and ideas going forward to create safe spaces for women and their families.

Women expressed the importance of continuity of care as a foundation for building a trusting relationship with their health providers before, during, and after their pregnancy. They highlighted that physical gestures of kindness and respect, as well as non-verbal emotional support, were just as important as verbal communication. Women valued health providers who were welcoming, smiled, held their hands, provided a safe space for questions, and proactively planned for the different pathways of care based on their individual needs and situations. These values were crucial in ensuring that women felt comfortable, supported, and respected during their interactions with health providers.

Women spoke about having open and honest conversations with their midwives about their experiences, concerns, and questions without judgement. They also appreciate when midwives take the time to explain procedures and treatments, involve them in decision-making, and provide information about available support services. Women also value midwives who understand their cultural and religious background and appreciate when midwives holistically approach their care and take into account their physical, emotional, and spiritual wellbeing. Additionally, women want midwives to be knowledgeable about the legal and ethical issues surrounding FGM and to be able to provide referrals to other healthcare providers as needed. The group was asked to roleplay in pairs, reflect on each other's scenarios, and consider whether they would change anything during their maternity care to improve it.

Participants described a supportive community environment in which educated healthcare providers delivered trauma-informed care linked to bicultural workers and local initiatives such as women's circles, buddying of new mothers with experienced mothers and community education that embraced them as unique individuals. Women provide several ideas to strengthen care at the systems level and across sectors. Suggestions included better coordination between maternity and child healthcare and other reproductive health needs before and after pregnancy.

Women described FGM-related physical and psycho-sexual healthcare needs that were poorly addressed and the need for proper referral and linked in health professionals. Comments were also raised concerning the legal system and that safeguarding efforts should also link women and girls into the health system early to receive counselling. Women highlighted the important role of health professionals in engaging men in conversations about FGM at the point of care and the need for advocacy to promote dialogue in the community to prevent FGM and support women. They suggest that health providers could engage in community campaigns and health education to raise awareness about FGM, its harmful effects, and available support services. By doing so, health providers could help break the silence around FGM and create a safe and supportive environment for FGM-affected women to access quality care and support. Safe spaces for women were highlighted and achievable through education, resource sharing, and access to health care, including physical and emotional support.

## 4 Discussion

The primary objective of this qualitative study was to examine the maternity care interactions Australian migrant women with FGM have had with healthcare providers and what women regard as quality interactions in line with woman-centred care. The research was conducted in two phases. The findings of the first phase were presented under three distinct categories of principles, enablers, and activities following Scholl et al. (27) framework. In phase two, narrative storytelling allowed women to share their stories that were represented in a visual format. This approach enabled rich data to be gathered to provide on women's experiences of care, their preferences, and how they believe health providers could better support them.

The findings from both phases demonstrate the usefulness of the dimensions across each domain of the Scholl et al. (27) model to articulate the crucial elements of women-centred maternity care for those affected by FGM. Women in our study clearly outlined important characteristics of providers that ensured a respectful, responsive, supportive, and collaborative relationship with a woman to ensure individualised care. While we did not identify a specific theme linked to the biopsychosocial aspect of Scholl et al. (27) model, this was integrated across these dimensions as evidenced by references to counselling access and culturally competent trauma-informed care. In terms of the dimensions across the enabler domain, quality communication between women and providers, support for physiological processes, access to care, coordination, and continuity of care reflected the main areas in the current model. The effectiveness of teams and team building did not emerge as a distinct theme; however, women did refer to positive experiences where their providers operated as a team. Women in the workshop identified the need to address the siloed nature of the healthcare system and report their desire to be served by effective teams with shared values across the reproductive health continuum. Dimensions across the activities domain were well represented in the analysis, with the involvement of family and friends integrated across both the interview and workshop data.

There may be unique factors of high-quality woman-centred care that are common to maternity care. A study involving a concept analysis of perinatal reviews identified seven shared features of quality obstetric and patient-centred care. These included respect and dignity, informed decision-making, therapeutic alliance, effective communication, consideration of social relationships and patient autonomy (29). In addition, the authors present five themes that they posit as unique to obstetrical care: continuity of care, privacy and confidentiality, provider education and status, the physical environment and equitable maternal care. Women in our study described positive experiences and quality care consistent with most of these themes except for privacy, confidentiality, and the physical environment. While these factors were not necessarily unimportant to women, they may have been well addressed in their care experiences or not considered a priority.

This study's findings, therefore, align with existing evidence indicating that women desire collaborative and interactive partnerships with competent healthcare providers. Respecting women's autonomy and involving them in the care process can empower women to actively engage in their care and make informed decisions (30). Empowerment can be achieved through the involvement of women in the co-production of their care to address their unique social, cultural, and economic needs, values and preferences. During the clinical encounter, for example, a woman's preferred level of involvement must be identified and choices explored, potentially with the use of decision aids or tools. In a shared decision-making approach, the clinician and woman act as partners, mutually exchanging information and deliberating on options to reach a consensus on the therapeutic course of action. The co-design of health professional education, tools, and guidance is critical to improving the quality and safety of maternity care, ensuring care satisfaction (31) and cost-effectiveness (32, 33). However, literature shows that patient involvement is not systematically integrated in clinical practice (34), nor have governments addressed woman-centred care in policies, as noted in research from Canada (35).

A scoping review of providing tools and guidelines for the care of women affected by FGM (16) found one guideline that integrated all the dimensions of patient-centred care from the Scholl model (36). However, this guidance is not focused on maternity care. No guidelines in this review specifically used a model of woman-centred care in their development and few identified the involvement of women in their creation. There is a need to co-design woman-centred care models to underpin guideline development to ensure high quality maternity care for women with FGM. Such research would also provide important empirical referents for the quality of maternity care.

The Scholl model could be adapted to develop woman-centred care guidelines to address specific needs, experiences and preferences of women with FGM. For instance, the dimensions of communication could be expanded to include specific reproductive health issues neglected during healthcare (37), such as FGM. The Scholl model does not explicitly address reproductive justice and may not take into account the social, economic, and political context in which reproductive decisions are made. These socio-cultural factors shape the norms, values and experiences of women and could be incorporated in the development of woman-centred care guidelines (38). Moreover, the model does not explicitly address trauma-informed care, an approach that recognises the impact of trauma on the health and wellbeing of patients, particularly relevant in the context of FGM (39).

The education of health providers, in addition to guidelines and tools, is a crucial element in quality care interactions. However, research has found that efforts are needed to improve how medical schools address patient-centred care in the curriculum. One study examining curricula from 16 medical schools in Canada found few documents noted or described patient-centred care or related concepts (40). While co-designed health professional guidelines that a woman-centred model of care underpins can provide women with a quality point-of-care interaction that enables shared decision-making (41), the wider context of a woman needs to be considered, as evidenced by the workshop findings. Care interactions need to be delivered within an ecosystem with community support to ensure women are health-literate and empowered to engage in their care (42). Community outreach workers, health promotion activities, and access to language support services are critical (43, 44).

This study is one of its first kind to examine woman-centred care maternity care for women with FGM that utilised both individual interviews and an active workshop that allowed a comprehensive understanding of the point-of-care experiences and needs of women across the domains of the Scholl et al. (27) model of patient-centred care. The results may not be representative of the experiences of all women with FGM who have received maternity services in Australia, as this study was only conducted with participants who were all from certain areas of Sydney. The findings of this study may not be generalisable to other contexts or countries with different healthcare systems and cultural norms. Additionally, the use of purposive sampling may have led to self-selection bias, where only those women who were interested in the topic or had positive experiences may participate in the study. The interviews and workshop were conducted in English, which may have limited the participation of women who did not speak English fluently.

## 5 Conclusion

This study suggests that a model of patient-centredness is useful for identifying quality point-of-care maternity interactions for women with FGM. The provision of woman-centred care requires a comprehensive multidisciplinary approach that considers the diverse needs and experiences of women, particularly those who are marginalised or face discrimination. Therefore, besides the inputs from experts and clinicians, we need to engage community members and women in the design of education, tools and guidelines to ensure they are genuinely woman-centred.

## Data Availability

The datasets presented in this article are not readily available because the original dataset used in this study contains confidential and sensitive information about women who have undergone Female Genital Mutilation (FGM) and their experiences of maternity care in Australia. Due to the nature of the data and the need to protect the privacy and confidentiality of the participants, there are restrictions that apply to the dataset. Requests to access the datasets should be directed to sabera.turkmani@uts.edu.au.

## References

[1] Fontein-Kuipers Y de Groot R van Staa A. Woman-centered care 2.0: bringing the concept into focus. Eur J Midwifery. (2018) 2:5. 10.18332/ejm/91492 PMC7846029 33537566

[2] Leap N. Woman-centred or women-centred care: does it matter? Br J Midwifery. (2009) 17(1):12–6. 10.12968/bjom.2009.17.1.37646

[3] Brady S Lee N Gibbons K Bogossian F. Woman-centred care: an integrative review of the empirical literature. Int J Nurs Stud. (2019) 94:107–19. 10.1016/j.ijnurstu.2019.01.001 30951986

[4] Hong H Oh HJ. The effects of patient-centered communication: exploring the mediating role of trust in healthcare providers. Health Commun. (2020) 35(4):502–11. 10.1080/10410236.2019.1570427 30706741

[5] Abdulcadir J Catania L Hindin MJ. Female genital multilation. A visual reference and learning tool for health care professionls. Obstet Gynecol. (2016) 128:958–63. 10.1097/AOG.0000000000001686 27741194

[6] UNFPA, WHO, ICM. The State of the Worlds’s Midwifery 2021. (2021). Available at: https://www.unfpa.org/sites/default/files/pub-pdf/21-038-UNFPA-SoWMy2021-Report-ENv4302.pdf

[7] Australian Government. Person-Centred Care ACT: The Australian Commission on Safety and Quality in Health Care. (2022). Available at: https://www.safetyandquality.gov.au/our-work/partnering-consumers/person-centred-care

[8] World Health Organization. Care of Girls and Women Living with Female Genital Mutilation: A Clinical Handbook. Geneva: World Health Organization (WHO) (2018). Available at: https://apps.who.int/iris/bitstream/handle/10665/272429/9789241513913-eng.pdf?ua=1

[9] Ahmed S Lee S Shommu N Rumana N Turin T. Experiences of communication barriers between physicians and immigrant patients: a systematic review and thematic synthesis. Patient Exp J. (2017) 4(1):122–40. 10.35680/2372-0247.1181

[10] Dawson A Turkmani S Varol N Nanayakkara S Sullivan E Homer CS. Midwives’ experiences of caring for women with female genital mutilation: insights and ways forward for practice in Australia. Women Birth. (2015) 28(3):207–14. 10.1016/j.wombi.2015.01.007 25686876

[11] Dawson A Homer CS Turkmani S Black K Varol N. A systematic review of doctors’ experiences and needs to support the care of women with female genital mutilation. Int J Gynaecol Obstet. (2015) 131(1):35–40. 10.1016/j.ijgo.2015.04.033 26118329

[12] Turkmani S Homer CS Dawson A. Understanding the experiences and needs of migrant women affected by female genital mutilation using maternity services in Australia. Int J Environ Res Public Health. (2020) 17(5):1491. 10.3390/ijerph17051491 32110898 PMC7084919

[13] Evans C Tweheyo R McGarry J Eldridge J Albert J Nkoyo V Seeking culturally safe care: a qualitative systematic review of the healthcare experiences of women and girls who have undergone female genital mutilation/cutting. BMJ Open. (2019) 9(5):e027452. 10.1136/bmjopen-2018-027452 31147364 PMC6549627

[14] Evans C Tweheyo R McGarry J Eldridge J Albert J Nkoyo V Crossing cultural divides: a qualitative systematic review of factors influencing the provision of healthcare related to female genital mutilation from the perspective of health professionals. PLoS One. (2019) 14(3):e0211829. 10.1371/journal.pone.0211829 30830904 PMC6398829

[15] Dawson A Turkmani S Fray S Varol N Homer CS. Evidence to inform education, training and supportive work environments for midwives involved in the care of women with female genital mutilation: a review of global experience. Midwifery. (2015) 31(1):229–38. 10.1016/j.midw.2014.08.012 25246318

[16] Dawson A Assifi A Turkmani S. Woman and girl-centred care for those affected by female genital mutilation: a scoping review of provider tools and guidelines. Reprod Health. (2022) 19(1):50. 10.1186/s12978-022-01356-3 35193606 PMC8862274

[17] Kent and Medway Council. Kent and Medway Female Genital Mutilation Operational Guidelines. Kent Safeguarding Children Board, Medway Safeguarding Children Board, and the Kent and Medway Safeguarding Adults Board. (2018).

[18] Bracknell Forest Council. Female Genital Mutilation (Fgm) Referral to the Multi-Agency Safeguarding Hub (MASH) Bracknell Forest: Bracknell Forest Council. (2017). Available at: https://health.bracknell-forest.gov.uk/jsna/people-and-places/vulnerable-groups/female-genital-mutilation-fgm/

[19] Gagliardi AR Green C Dunn S Grace SL Khanlou N Stewart DE. How do and could clinical guidelines support patient-centred care for women: content analysis of guidelines. PLoS One. (2019) 14(11):e0224507. 10.1371/journal.pone.0224507 31703076 PMC6839851

[20] Perron L Senikas V Burnett M Davis V. Female genital cutting. J Obstet Gynaecol Can. (2013) 35(11):e1–e18. 10.1016/S1701-2163(15)30792-1 32007263

[21] England PH. Understanding Female Genital Mutilation (FGM) Helping Health Visitors and School Nurses Tackle Female Genital Mutilation. London: Public Health England (2016).

[22] Department of Health and Community Services. Female Genital Mutilation Resource Manual for Health Professionals. Darwin, Northern Territory: Women’s Health Strategy Unit, Dept. of Health (2006).

[23] Chandra-Mouli V Camacho AV Michaud PA. WHO guidelines on preventing early pregnancy and poor reproductive outcomes among adolescents in developing countries. J Adolesc Health. (2013) 52(5):517–22. 10.1016/j.jadohealth.2013.03.002 23608717

[24] Hajizadeh K Vaezi M Meedya S Charandabi SMA Mirghafourvand M. Designing a respectful maternity care guideline: a multiphase study. Reprod Health. (2022) 19(1):81. 10.1186/s12978-022-01389-8 35346250 PMC8961910

[25] Smits DW Kv M Klem M Alsem M Ketelaar M. Designing a tool to support patient and public involvement in research projects: the involvement matrix. Res Involv Engagem. (2020) 6(1):30. 10.1186/s40900-020-00188-4 32550002 PMC7296703

[26] Kyngäs H Kaakinen P. Deductive content analysis. In: Kyngäs H Mikkonen K Kääriäinen M, editors. The Application of Content Analysis in Nursing Science Research. Cham: Springer International Publishing (2020). p. 23–30.

[27] Scholl I Zill JM Härter M Dirmaier J. An integrative model of patient-centeredness–a systematic review and concept analysis. PLoS One. (2014) 9(9):e107828. 10.1371/journal.pone.0107828 25229640 PMC4168256

[28] Whitney C Evered JA. The qualitative research distress protocol: a participant-centered tool for navigating distress during data collection. Int J Qual Methods. (2022) 21:16094069221110317. 10.1177/16094069221110317

[29] Dong K Jameel B Gagliardi AR. How is patient-centred care conceptualised in obstetrical health? Comparison of themes from concept analyses in obstetrical health- and patient-centred care. Health Expect. (2022) 25(3):823–39. 10.1111/hex.13434 35026046 PMC9122412

[30] Green JM. Integrating women’s views into maternity care research and practice. Birth. (2012) 39(4):291–5. 10.1111/birt.12003 23281947

[31] Payne JM D'Antoine HA France KE McKenzie AE Henley N Bartu AE Collaborating with consumer and community representatives in health and medical research in Australia: results from an evaluation. Health Res Policy Syst. (2011) 9(1):18. 10.1186/1478-4505-9-18 21569591 PMC3118959

[32] Pickles J Hide E Maher L. Experience based design: a practical method of working with patients to redesign services. Clin Gov. (2008) 13:51–8. 10.1108/14777270810850634

[33] Ovretveit J. Does Improving Quality Save Money? A Review of Evidence of which Improvements to Quality Reduce Costs to Health Service Providers: The Health Foundation. (2009). Available at: https://www.health.org.uk/sites/default/files/DoesImprovingQualitySaveMoney_Evidence.pdf

[34] Miller T Reihlen M. Assessing the impact of patient-involvement healthcare strategies on patients, providers, and the healthcare system: a systematic review. Patient Educ Couns. (2023) 110:107652. 10.1016/j.pec.2023.107652 36804578

[35] Gagliardi AR Dunn S Foster AM Grace SL Khanlou N Stewart DE Is patient-centred care for women a priority for policy-makers? Content analysis of government policies. Health Res Policy Syst. (2020) 18(1):23. 10.1186/s12961-020-0533-z 32070365 PMC7029558

[36] Coho C Parra S Hussein L Laffy C. Female Genital Trauma: Guidelines for Working Therapeutically with Survivors of female genital mutilation. London: Dhalia Project, The Centre for Psychosexual Health, National Council of Pschotherapists (2019). Available at: https://manorgardenscentre.org/resources/1924_Female_Genital_Trauma_Report_Web.pdf

[37] The American College of Obstetricians and Gynecologists. Effective patient–physician communication. Obstet Gynecol (New York 1953). (2014) 123(2, PART 1):389–93. 10.1097/01.AOG.0000443279.14017.12 24451677

[38] Kirkendall A Dutt A. Refugee women’s pregnancy and childbirth experiences in the US: examining context through a reproductive justice framework. Fem Psychol. (2023) 33(4):529–49. 10.1177/09593535221149166

[39] Lurie JM Pilato T Kaur G. Female genital mutilation/cutting and birthing: enhanced education and training is critical for health care providers. J Glob Health. (2022) 12:03059. 10.7189/jogh.12.03059 36056805 PMC9440617

[40] Anderson NN Gagliardi AR. Unclear if future physicians are learning about patient-centred care: content analysis of curriculum at 16 medical schools. Med Teach. (2021) 43(9):1085–91. 10.1080/0142159X.2021.1918332 33915064

[41] Ebert L Bellchambers H Ferguson A Browne J. Socially disadvantaged women’s views of barriers to feeling safe to engage in decision-making in maternity care. Women Birth. (2014) 27(2):132–7. 10.1016/j.wombi.2013.11.003 24355713

[42] Higginbottom GMA Hadziabdic E Yohani S Paton P. Immigrant women’s experience of maternity services in Canada: a meta-ethnography. Midwifery. (2014) 30(5):544–59. 10.1016/j.midw.2013.06.004 23948185

[43] Shaw D Guise J-M Shah N Gemzell-Danielsson K Joseph KS Levy B Drivers of maternity care in high-income countries: can health systems support woman-centred care? Lancet. (2016) 388(10057):2282–95. 10.1016/S0140-6736(16)31527-6 27642026

[44] Barnett R. A Framework for Women Centred Health Vancouver Vancouver/Richmond Health Board. (2001). Available at: http://www.atira.bc.ca/AdvancingHealthCareWorkshop/womenframework.pdf

